# Instrument for Arresting Epistaxis

**Published:** 1851-06

**Authors:** 


*Instrument for arresting Epistaxis.* By M. Gariel.—This is a tube made of caoutchouc, carrying at its extremity a dilatable balloon, which, when introduced into the nostrils in its undistended state, may, by the process of insufflation, be made to assume such dimensions, and exerting such pressure as com; letely to arrest the hemorrhage.

A simple method of plugging the posterior nares suggests itself in examining this tube with dilatable extremity. This operation as at present performed, whether with a special apparatus, or with an ordinary catheter, is frequently very troublesome, though simple in appearance. If the tube be introduced from before backwards through the cavities of the nose, until it has quite cleared the posterior nares and arrived in the pharynx, and be then dilated and drawn forwards, we obtain a more complete and manageable plug than that usually made of lint.—*Dublin Quarterly Journal of Med. Science.*

